# Modulation of Protein Quality Control and Proteasome to Autophagy Switch in Immortalized Myoblasts from Duchenne Muscular Dystrophy Patients

**DOI:** 10.3390/ijms19010178

**Published:** 2018-01-07

**Authors:** Marion Wattin, Loïc Gaweda, Pascale Muller, Mathieu Baritaud, Charlotte Scholtes, Chloé Lozano, Kathrin Gieseler, Carole Kretz-Remy

**Affiliations:** Institut NeuroMyoGène, CNRS UMR5310, INSERM U1217, Université Lyon 1, F-69622 Villeurbanne, France; Université de Lyon, Lyon, France; marion.wattin@univ-lyon1.fr (M.W.); loic.gaweda@etu.univ-lyon1.fr (L.G.); pascale.muller@univ-lyon1.fr (P.M.); mathieu.baritaud@gmail.com (M.B.); charlotte.scholtes@univ-lyon1.fr (C.S.); chloe.lozano@etu.univ-lyon1.fr (C.L.); kathrin.gieseler@univ-lyon1.fr (K.G.)

**Keywords:** protein quality control, protein aggregation, chaperones, autophagy, proteasome, BAG3, HSPB8, NFκB, DMD, muscle

## Abstract

The maintenance of proteome integrity is of primary importance in post-mitotic tissues such as muscle cells; thus, protein quality control mechanisms must be carefully regulated to ensure their optimal efficiency, a failure of these processes being associated with various muscular disorders. Duchenne muscular dystrophy (DMD) is one of the most common and severe forms of muscular dystrophies and is caused by mutations in the dystrophin gene. Protein quality control modulations have been diversely observed in degenerating muscles of patients suffering from DMD or in animal models of the disease. In this study, we investigated whether modulations of protein quality control mechanisms already pre-exist in undifferentiated myoblasts originating from DMD patients. We report for the first time that the absence of dystrophin in human myoblasts is associated with protein aggregation stress characterized by an increase of protein aggregates. This stress is combined with BAG1 to BAG3 switch, NFκB activation and up-regulation of BAG3/HSPB8 complexes that ensure preferential routing of misfolded/aggregated proteins to autophagy rather than to deficient 26S proteasome. In this context, restoration of pre-existing alterations of protein quality control processes might represent an alternative strategy for DMD therapies.

## 1. Introduction

The maintenance of proteome integrity is of great importance for cell viability. Indeed, proteins must fold into specific three-dimensional structures to acquire their functional native states, which is in precarious equilibrium. Actually, proteins often misfold as a result of stochastic fluctuations, mutations or environmental stresses such as elevated temperatures, exposure to chemicals, production of reactive oxygen species or physiological aging [1,2,3]. Because of their altered spatial arrangement, misfolded proteins expose hydrophobic domains that drive protein aggregation. Misfolded/aggregated proteins can sequester other functional cellular components, leading to cell cytotoxicity; this phenomenon is considered a major mechanism of human diseases called “protein conformational diseases” [1]. Consequently, cells possess protein quality control (PQC) machinery that preserves the health of its proteome by three main strategies: refolding, sequestration or degradation. Refolding is achieved by molecular chaperones that bind to non-native proteins [4]. Chaperones can be classified according to their mode of action: (i) the foldases such as HSPA family that assist protein refolding in an ATP-dependent way; and (ii) the ATP-independent holdases, such as HSPB1, HSPB5 or HSPB8, which bind partially unfolded client proteins and, thus, protect them from aggregation [5]. Chaperones are associated with co-chaperones and form a dynamic network that can adapt to the environment, as for example, when refolding is not possible the association of BAG1 or BAG3 co-chaperones to HspA1/A8 can redirect misfolded/aggregated proteins to degradation mechanisms; the proteasome for the former and the autophagy for the latter [6]. Degradation is achieved by two major pathways: the ubiquitin-proteasome system (UPS) and autophagy. The 26S proteasome is a barrel-like structure containing catalytic β-subunits bearing caspase-like, trypsine-like and chymotrypsin-like proteolytic activities [7,8]. Substrate proteins are targeted to the proteasome through multi-ubiquitination accomplished by E1, E2 and E3 enzymes [9] and are degraded into small peptides [10]. Macroautophagy (herein referred as autophagy) is a self-eating process starting with de novo formation of double-membrane autophagosomes that engulf parts of the cytoplasm and fuse with acidic lysosomes to form autolysosomes [11,12]; the autophagosome content is then degraded by lysosomal hydrolases and released for recycling. The autophagic process is regulated by ATG proteins and requires the ULK1 kinase complex, the interaction of beclin1 with class III phosphatidylinositol 3-kinase (PI3K), the ATG9 cycling system, the WIPIs, and the Atg12-Atg5 and the LC3-phosphatidylethanolamine (LC3-II) conjugation systems, which are under the control of various signal transduction pathways [13,14,15]. Autophagy has long been described as a bulk degradation process which allows energy salvage after starvation for example [16]. However, it is now abundantly described that autophagy can selectively degrade cytoplasmic components such as protein aggregates by targeting cargos to the autophagosomal membrane protein LC3-II, via the use of autophagic receptors and adaptors (such as p62 or BAG3) [17,18].

When PQC mechanisms are inefficient or overwhelmed, protein aggregates are formed and accumulate, leading eventually to protein conformational diseases such as neurodegenerations or muscular disorders, of which muscular dystrophies are characterized by progressive wasting of skeletal muscles [19,20]. Duchenne muscular dystrophy (DMD) is one of the most common forms of muscular dystrophies (one out of 3500 new-born males) [21,22]. DMD is characterized by progressive muscle degeneration starting in early childhood and affecting cardiac and skeletal muscles such as diaphragm [23]. This X-linked recessive disorder is caused by duplications, deletions or point mutations of the human dystrophin gene [24]. The dystrophin protein is essential for maintenance of muscle cell membranes; it recruits other structural and signaling proteins to the sarcolemma, allowing the formation of the dystrophin-associated glycoprotein complex that plays a key role during muscle contraction but also scaffolds intracellular signaling proteins [25]. In addition to its role in differentiated muscles, it was recently reported that dystrophin is necessary for maintaining the asymmetric division and the regenerating potential of mouse satellite cells [26]. To date, no cure for DMD has been discovered by researchers, but several pre-clinical and clinical settings are currently investigated [27]. Interestingly, some connections between PQC perturbation and altered muscles in DMD patients or in diverse animal models have been evidenced. For instance, an increase of molecular chaperones was observed in muscles of the *mdx* mouse model [28,29]; modulation of autophagy was also reported in *mdx* mice or muscle biopsies of DMD patients [25,30]. Moreover, our group also observed an increased number of autophagosomes in the nematode (*C. elegans*) DMD model and identified genetic suppressors of muscle degeneration connected to protein degradation pathways [31]. However, whether and how PQC contributes to dystrophin-dependent muscle degeneration is still unknown. In addition, all PQC studies on DMD models have been carried out on mature, degenerating muscles; it is thus still unknown whether PQC modulations pre-exist in DMD myoblasts, the myofibre precursors. We therefore performed an exhaustive study of PQC actors in a unique DMD model consisting of transformed human myoblast cell lines originating from dystrophic muscle biopsies of DMD patients. 

In this study, we observed an accumulation of intracellular protein aggregates in DMD myoblasts in comparison to controls. Moreover, we detected modulations of molecular chaperone levels, notably HSPB5 and HSPB8, a decreased activity of the 26S proteasome and a BAG1 to BAG3 molecular switch. These modulations of PQC processes were associated with an up-regulation of NFκB transcription factor activity, increased levels of autophagy actors (ATG3, LC3-II, HDAC6) and stimulation of the autophagic flux, but partial deficiency of autophagy maturation steps along with down-regulation of proteasome activity. These results demonstrate that PQC alterations pre-exist in DMD myoblasts and could represent interesting targets in palliative treatment strategies for DMD.

## 2. Results

### 2.1. Characterization of Immortalized Myoblasts Derived from Healthy Donors or Duchenne Muscular Dystrophy Patients

Since there is still no discovered cure for DMD, standardized tools that could allow the study of the molecular and cellular mechanisms involved in this disease are needed. In this context, human primary myoblasts derived from muscle biopsies of dystrophic patients have been used. However, these in vitro cultures suffer from phenotype variations and cellular senescence after prolonged cell division. The use of stable transformed cell lines deriving from human myoblasts isolated from muscular biopsies of DMD patients can overcome these drawbacks [32]. In this study, we used six immortalized myoblast cell lines expressing human telomerase reverse transcriptase (hTERT) and cyclin-dependent kinase-4 (CDK4), to study the involvement of protein quality control (PQC) in DMD pathology. W1 and W2 cell lines were obtained from biopsies of healthy donors whereas D1 to D4 cell lines were derived from muscle biopsies of DMD patients (see Materials and Methods).

**Figure 1:**
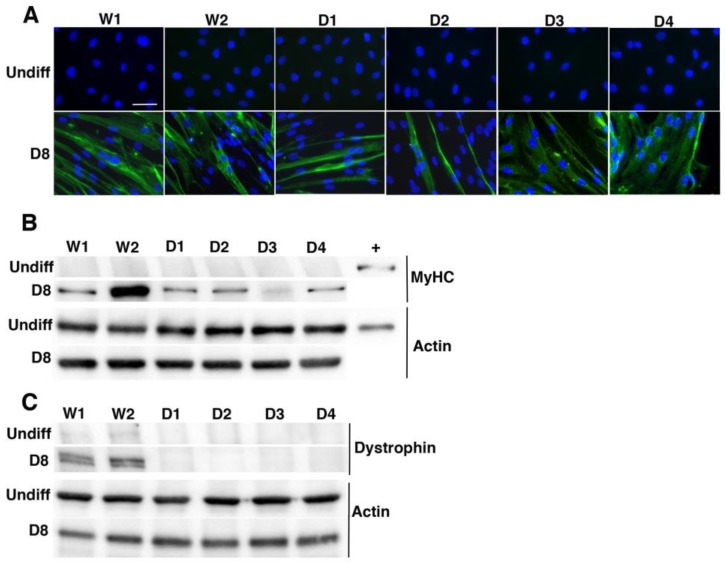
Characterization of human immortalized myoblast cell lines. (**A**) Wild Type (W1 & W2) and DMD (D1, D2, D3 & D4) cell lines were seeded on Matrigel-coated 35 mm dishes in proliferative medium. The day after, proliferative medium was replaced or not (Undiff) by differentiation medium. Cells were differentiated over eight days (D8), then myosin heavy chain (MyHC) immunostaining and Hoechst nucleus staining were performed (scale bar: 50 µm); (**B**,**C**) Total proteins of wild type and DMD cell lines were extracted and 10 μg were separated by SDS-PAGE. Analysis of MyHC (**B**) and dystrophin (**C**) expression was performed by immunoblotting thanks to the use of specific antibodies. Actin is used as a loading control. Results are representative of three independent experiments.

We first checked the myogenic signature and differentiation ability of the cells by detecting myosin heavy chains (MyHC) in control or DMD cell lines, during proliferation or differentiation conditions (Figure 1A,B). As seen by immunofluorescence analysis, after eight days of differentiation, all cell lines expressed MyHC, whereas undifferentiated cells did not (Figure 1A); moreover, at D8 we could distinguish fused myoblasts containing more than two nuclei in each cell line. These results were confirmed by Western blot analysis of MyHC levels. Indeed, MyHC was expressed in each cell line, albeit to a higher degree in W2 and to a lower degree in D3 cell line (Figure 1B), a heterogeneity that was already reported in various muscle models [33,34]. We then tested for dystrophin expression, by Western blot analysis using mandra1 antibody that recognizes the end of the C-terminal domain of dystrophin. We could detect low levels of dystrophin in proliferating/undifferentiated control cells (W1 and W2) but no expression in DMD cell lines (Figure 1C). In addition, as expected, we detected high levels of dystrophin in differentiated control cells but not in differentiated DMD cell lines. To confirm dystrophin expression in proliferating control myoblast cell lines, a Western blot analysis was performed with 30 μg of total protein extracts of control and DMD undifferentiated myoblasts and compared with the analysis 10 μg of total protein extracts from W2 differentiated control cell lines (Figure S1). Quantification of the dystrophin bands revealed a 13-fold higher level of dystrophin in differentiated myotubes compared to non-differentiated myoblasts from healthy donors; in contrast, no dystrophin band was detectable in protein extracts from DMD myoblasts or myotubes. Thus, our results demonstrate that dystrophin protein is expressed at the myoblast stage and consequently, DMD transformed myoblastic cell lines constitute a robust ex vivo model to assess the consequences of dystrophin absence on PQC mechanisms.

### 2.2. Protein Aggregation Is Increased in DMD Myoblasts

**Figure 2:**
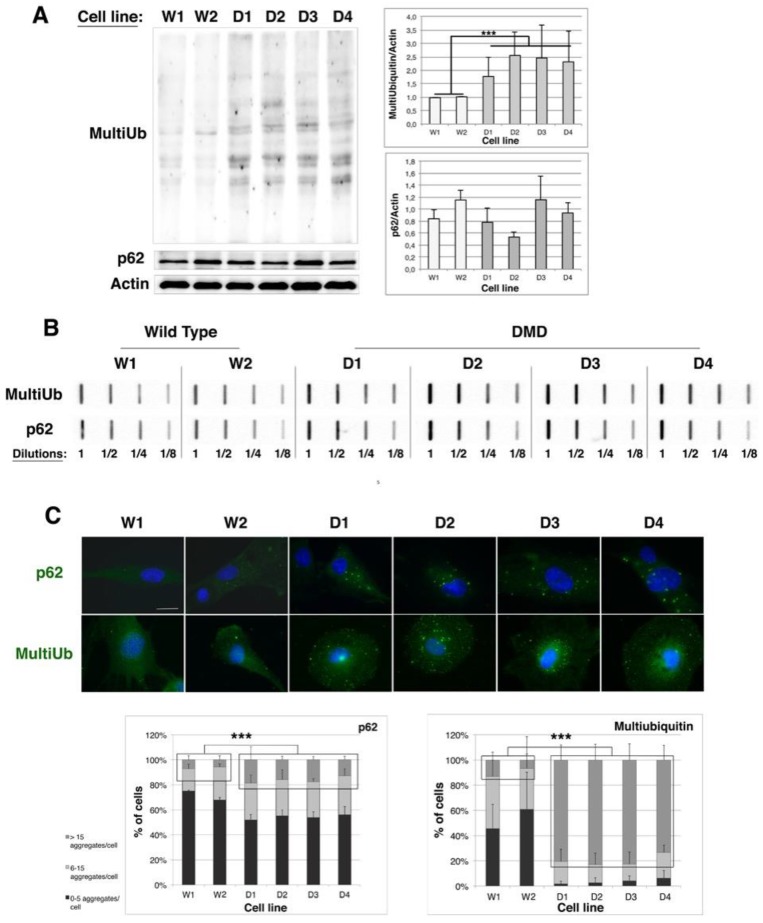
Protein aggregation is increased in human immortalized DMD myoblast cell lines. (**A**) 10 µg of total protein extracts of Wild Type (W1 & W2) and DMD (D1, D2, D3 & D4) cell lines were separated by SDS-PAGE and analyzed by immunoblotting, using specific antibodies directed against multi-ubiquitin, p62 or Actin (as a loading control). The histograms show means ± SD of normalized MultiUb/Actin and p62/Actin ratios (MultiUb/Actin or p62/Actin ratio of each cell line was divided by the mean of ratios of control cell lines (W1 and W2)); (*n* = 3 independent experiments); (**B**) Filter trap analysis: 2.5 µg of total protein extracts from WT and DMD cell lines were slot-blotted at four different dilutions (1, 1/2, 1/4 & 1/8) on a cellulose acetate membrane and probed with multi-ubiquitin and p62 antibodies. Result is representative of three independent experiments; (**C**) Immunostaining of multi-ubiquitin and p62 and Hoechst staining of nuclei were performed in Wild Type and DMD myoblasts (scale bar: 50 µm). The graph indicates the percentage of cells containing 0–5, 6–15 and over 15 aggregates (green dots) larger than 2 µm. Counting of aggregates was performed twice on 100 cells per cell line, in three independent experiments. *** *p* < 0.001, one way ANOVA parametric test.

To evaluate the involvement of protein quality control in Duchenne muscular dystrophy, we first analyzed the protein aggregation status of control (W1 and W2) and DMD (D1–D4) immortalized myoblast cell lines. To this end, we focused on multi-ubiquitinated proteins or p62 protein that are markers of protein aggregates and are involved in proteasome or autophagy addressing, respectively [35,36]. As shown in Figure 2A (Western blot and quantifications), in DMD cell lines the level of multi-ubiquitinated proteins was increased by 1.7 (D1) to 2.4-fold (D2) in comparison to control ones. However, no significant modulation of p62 level was detected. We next determined the protein aggregation status of multi-ubiquitinated proteins or p62-associated aggregates by filter trap analysis (Figure 2B). Four different dilutions of total protein extracts from each cell line were slot-blotted onto a nitrocellulose membrane. Soluble proteins passed through the membrane whereas protein aggregates were trapped on the membrane and were immunodetected with antibodies against multiubiquitin or p62. For filter trap revealed with anti-multi-ubiquitin, we observed that the 1/8 dilution of D1 extract was of similar intensity than the 1/4 dilution of W1 and W2 protein extracts; the same observation was performed with the 1/8 dilution of D2 extracts. At last 1/8 dilutions of D3 and D4 extracts had a transitional intensity between the 1/4 and 1/2 dilutions of W1 and W2 extracts. We could thus conclude that there was a two-fold (D1 and D2) to three-fold (D3 and D4) increased level of protein aggregates conjugated to multi-ubiquitin in DMD cell lines compared to control cells. Similarly, the 1/8 dilutions of D1–D4 extracts were of identical intensities than the 1/4 dilutions of W1 and W2 extracts, allowing us to conclude to a two-fold increase of p62-containing aggregates in DMD cell lines as compared to control cells. Those quantifications were validated by densitometric analysis of filter traps (Table S1). In addition, these results were confirmed by immunofluorescence analysis, in which more aggregates labeled by multi-ubiquitin or p62 antibodies were detected in DMD cell lines than in controls (Figure 2C). Quantification of aggregates revealed that the percentage of cells containing more than fifteen p62-containing aggregates is increased by 2.5-fold and the percentage of cells containing more than 15 multi-ubiquitinated aggregates by 13-fold, in DMD cell lines with respect to controls. Taken together, our results indicate an increased protein aggregation level in DMD immortalized myoblast cell lines in comparison to their control counterparts, which suggests that protein quality control might be less efficient in myoblasts derived from muscle biopsies of DMD patients than from healthy donors.

### 2.3. HSPB5 and HSPB8 Levels Are Modulated in DMD Myoblasts

Heat shock proteins (HSP) are sensors of protein misfolding/aggregation and help the proteins to recover their native conformation. Since we observed increased protein aggregation in DMD immortalized myoblast cell lines, we asked whether HSPs were efficient in these cells.

**Figure 3:**
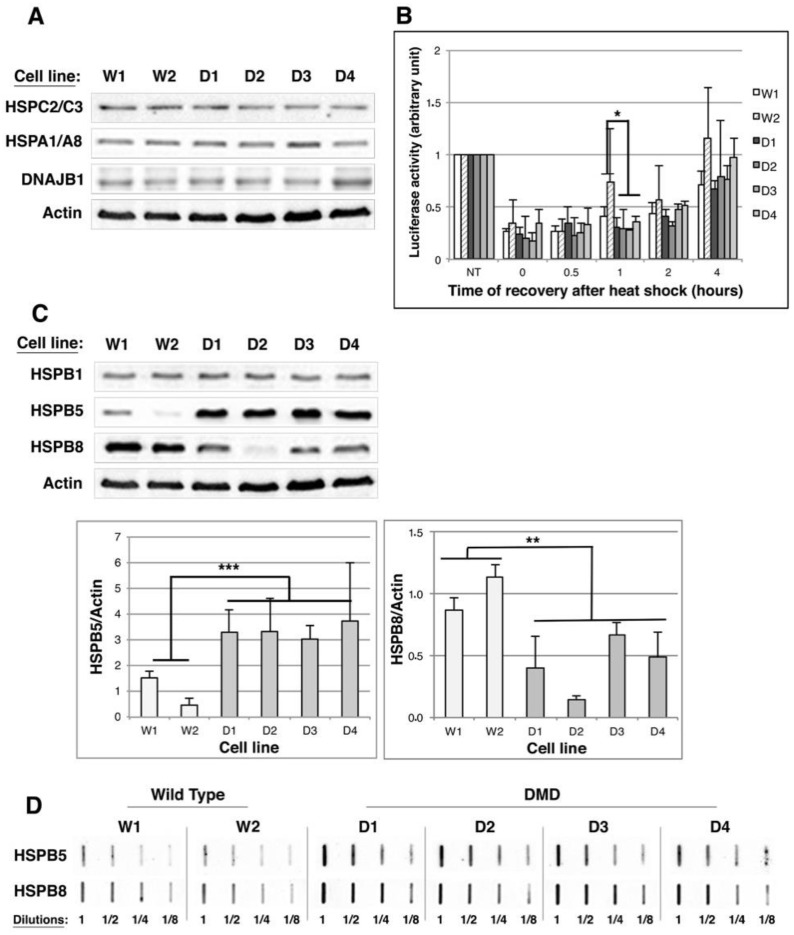
HSPB5 and HSPB8 levels are modulated in human immortalized DMD myoblast cell lines; (**A**) 10 µg of total protein extracts of Wild Type (W1 & W2) and DMD (D1 to D4) cell lines were separated by SDS-PAGE. Heat Shock proteins expression was analyzed by immunoblotting, using anti-HSPC2/C3, -HSPA1/A8, -DNAJB1 and -Actin (as a loading control); (**B**) Luciferase refolding assay. Control and DMD cell lines were transiently transfected with pGL3-promotor vector. The day after, they were submitted or not (NT) to a 30-min heat-shock treatment at 43 °C followed by 0–4 h of recovery at 37 °C before quantification of luciferase activity (see Material and Methods). NT conditions of each cell line were set at 1; (**C**)—As in (**A**) but Western-blots were hybridized with HSPB1, B5 and B8 antibodies. Actin was used as a loading control. The histograms indicate the means ± SD of normalized HSPB5/Actin and HSPB8/Actin ratios as described in Figure 2; (**D**) Total proteins of Wild Type and DMD cell lines were extracted and 2.5 μg of proteins were submitted to filter trap analysis as described in Figure 2. Membranes were hybridized with anti-HSPB5 and anti-HSPB8 antibodies. Results are representative of three independent experiments. * *p* < 0.05, ** *p* < 0.01, *** *p* < 0.001, one way ANOVA parametric test.

We first focused on foldases and determined the level of their major members that are HSPC2/C3 (Hsp90α and β), HSPA1/A8 (Hsp70/Hsc70) and DNAJB1 (Hsp40). As shown in Figure 3A, no modulation of the major foldase levels could be detected by Western blot analysis in control or DMD myoblasts; this observation was confirmed by statistical analysis of quantifications (Figure S2A). We then determined the global folding activity in each of the control and DMD cell lines by performing a refolding luciferase assay (Figure 3B). This assay allows determining the cell-folding capacity by quantifying cell extract ability to restore the enzymatic activity of constitutively expressed luciferase that has been inhibited by heat shock-induced denaturation. We observed that the kinetics of luciferase activity recovery after the heat shock was similar in all cell lines, indicating that the folding capacity of DMD myoblastic cell lines was not altered.

We next analyzed by Western blot the levels of the major muscle holdases: HSPB1 (Hsp27), HSPB5 (αB-crystallin) and HSPB8 (Hsp22). No modulation of HspB1 level was observed between control and DMD cell lines (Figure 3C and Figure S2B). By contrast, a two- to seven-fold increase of HSPB5 level and a 20–78% decrease of HSPB8 level were observed in DMD cell lines in comparison to control cells (Figure 3C). We therefore asked whether HspB5 and HspB8 could be recruited to protein aggregates by filter trap analysis. With HspB8 filter trap (Figure 3D and Table S1), we observed that the 1/8 dilutions of D1–D4 protein extracts were revealed with similar intensities than the 1/4 or 1/2 dilutions of W1 and W2 protein extracts, indicating a two- to four-fold increase of HSPB8 recruitment to protein aggregates in DMD cell lines, whereas its global expression is decreased in these cells. Thus, these results suggest that HSPB8 is specifically redirected to protein aggregates in DMD myoblasts. As for HspB5 filter trap, 1/8 dilutions of DMD protein extracts were revealed with similar intensities than 1/2 dilutions of control (W1 and W2) protein extracts, indicating a four-fold increase of HSPB5-containing aggregates in DMD cell lines compared to control cell lines (Figure 3D); this indicates that HSPB5 overexpression goes along with its recruitment to protein aggregates.

Since a major function of HSPB5 is the chaperoning of cytoskeleton filaments [37,38,39], we analyzed by immunofluorescence actin microfilaments, microtubules, or vimentin intermediate filaments in control and DMD myoblasts. However, we did not detect any modification of actin bundles, vimentin or microtubule networks in DMD cell lines compared to controls (Figure S3).

In conclusion, our results indicate that the refolding capacity of DMD cell lines is not altered; however, the expression of HSPB5 and HSPB8 holdases is modulated, these proteins being preferentially redirected to protein aggregates; this observation might suggest that the chaperone network in DMD myoblasts is harnessed but overwhelmed.

### 2.4. Proteasome Activity Is Decreased in DMD Myoblasts

**Figure 4:**
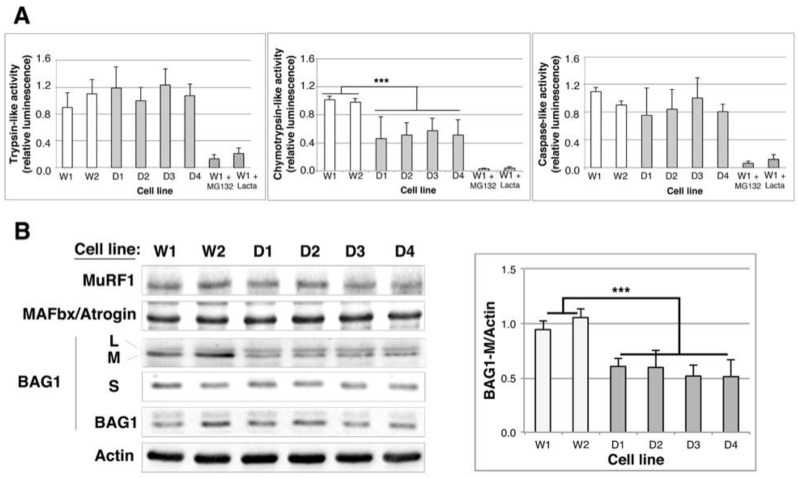
UPS efficiency is altered in human immortalized DMD myoblast cell lines. (**A**) Trypsine-, chymotrypsin- and caspase-like activities of the 26S proteasome were quantified by using specific fluorogenic substrates, in control (W1 and W2) and DMD (D1–D4) cell lines. As a negative control, two proteasome inhibitors, MG132 and lactacystin (Lacta) were added to the cell culture medium (2 h, 10 µM). Data were normalized to the mean of the Wild Type results. Statistical analyses (ANOVA test, *n* = 4) show a significant decrease of chymotrypsin-like activity in DMD cell lines; (**B**) 10 μg of total protein extracts of Wild Type and DMD cell lines were separated by SDS-PAGE. Analysis of MuRF1, MAFbx/Atrogin and BAG1 isoforms expression was performed using specific antibodies. Actin is revealed as a loading control. The histogram shows the means ± SD of normalized BAG1M/Actin ratios as described in Figure 2 (*n* = 3). *** *p* < 0.001, one way ANOVA parametric test.

We then asked whether degradation mechanisms might be roped in for the clearance of protein aggregates in DMD myoblasts. As for UPS, the increase of multi-ubiquitinated and/or aggregated protein levels observed in DMD cell lines could be the consequence of a modulation of (i) the ubiquitination of misfolded proteins, (ii) the targeting of misfolded and ubiquitinated proteins to the UPS or (iii) the enzymatic activity of the proteasome. We first quantified enzymatic activity of 26S proteasome by incubating WT and DMD myoblasts with luminogenic substrates specific for chymotrypsin-, trypsin- and caspase-like activities. Trypsin- and caspase-like activities were not modified between control and DMD cell lines (Figure 4A); in contrast, we observed a 50% decrease of chymotrypsin-like activity. We then checked the expression of the major muscle-ubiquitin E3 ligases that are MuRF1 (muscle really interesting novel gene (RING) finger-1) and MAFbx/Atrogin-1 (muscle atrophy F-box) by Western blot analysis. MuRF1 and MAFbx/Atrogin-1 levels were identical in control and DMD myoblasts suggesting that ubiquitination efficiency is similar in these cell lines (Figure 4B and Figure S4). At last, we quantified the BAG1 co-chaperone level, which is involved in targeting multi-ubiquitinated proteins to the proteasome. BAG1 exists as four isoforms; we observed a 45% decrease of the medium isoform of BAG1 (BAG1-M) in DMD myoblasts as compared to controls; by contrast, BAG1L, BAG1 and BAG1S levels were unmodified (Figure 4B). 

Taken together, our results indicate that the addressing of multi-ubiquitinated proteins to the proteasome mediated by BAG1 might be less efficient in DMD cells; in addition, as in these cells the chymotrypsin-like activity of the 26S proteasome activity is decreased, we propose that misfolded/aggregated protein degradation by the proteasome is impaired in DMD myoblasts.

### 2.5. BAG1/BAG3 Ratio Is Inverted, BAG3/HSPB8 Complexes Are Increased and Autophagy Is Up-Regulated in DMD Myoblasts

**Figure 5:**
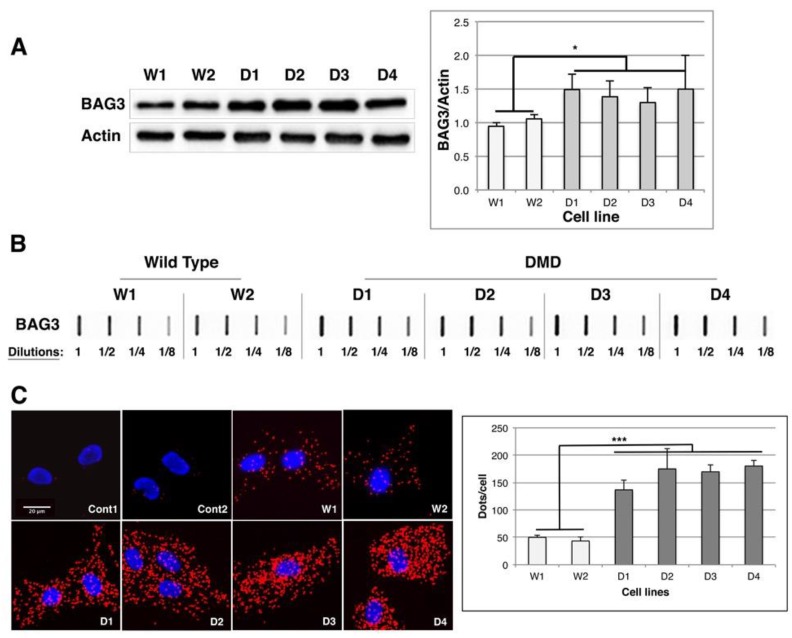
BAG3 level and BAG3/HSPB8 complexes are up-regulated in DMD myoblasts. (**A**) 10 μg of total protein extracts of Wild Type (W1 & W2) and DMD (D1 to D4) cell lines were separated by SDS-PAGE and analyzed by immunoblots probed with BAG3 and Actin antibodies. The histogram shows the means ± SD of normalized BAG3/Actin ratios as described in Figure 2 (*n* = 3); (**B**) 2.5 μg of total protein extracts of Wild Type and DMD cell line were submitted to filter trap analysis as described in Figure 2. Membranes were hybridized with anti-BAG3 antibody. Results are representative of four independent experiments; (**C**) Control and DMD cell lines were fixed, permeabilized and submitted to proximity ligation assay for revealing BAG3/HSPB8 complexes (red dots) along with DAPI staining of nuclei (blue). The graph shows the means ± SD of the quantification of PLA dots/cell (*n* = 3). * *p* < 0.05, *** *p* < 0.001, one way ANOVA parametric test.

In an aggregation-prone environment a reciprocal change in the expression of BAG1 and BAG3 co-chaperone was described, which enhances the autophagy-lysosome degradation pathways [40,41]. We thus quantified BAG3 levels by Western blot in control and DMD cell lines and observed a 1.3 (D3) to 1.5-fold (D1 and D4) up-regulation of BAG3 levels in DMD cell lines in comparison to controls (Figure 5A). Moreover, in filter trap analysis the 1/8 dilutions of DMD protein extracts were revealed with similar intensity than the 1/4 dilutions of W1 and W2 protein extracts, indicating a twofold increase of BAG3-containing aggregates in comparison to control cells (Figure 5B and Table S1); thus, BAG3 up-regulation seems to go along with its recruitment to protein aggregates. Since we and others demonstrated that BAG3 in complex with HSPB8 could be recruited to protein aggregates and involved in the selective clearance of protein aggregates by autophagy [17,42,43,44], we quantified BAG3/HSPB8 complexes in control and DMD cell lines by proximity ligation assay (PLA, Figure 5C). This method allows the detection of molecules that interact or are in very close proximity by using oligonucleotide labeled antibodies binding to two proteins in a complex (see Materials and Methods). PLA detection of endogenous BAG3/HSPB8 complexes generated a more abundant signal in DMD cell lines than in control cells; indeed, the average number of dots (representing a single protein complex) per cell was increased by 2.7 (D1) to 4.5-fold (D4) in DMD myoblasts compared to controls (Figure 5C, graph). In addition, there was no signal in negative control experiments performed with only one antibody (anti-BAG3, Cont1) or with antibodies for BAG3 and PML (located in the cytoplasm and the nucleus, respectively) (Cont2). Our results thus indicate that there is a switch between BAG1 and BAG3 levels in DMD cell lines associated with an up-regulation of the level of BAG3/HSPB8 complexes, suggesting that selective autophagic process could be stimulated in order to degrade protein aggregates.

**Figure 6:**
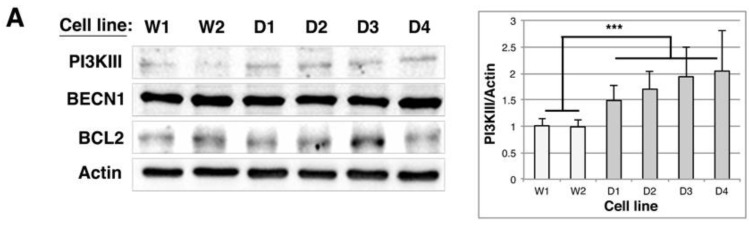

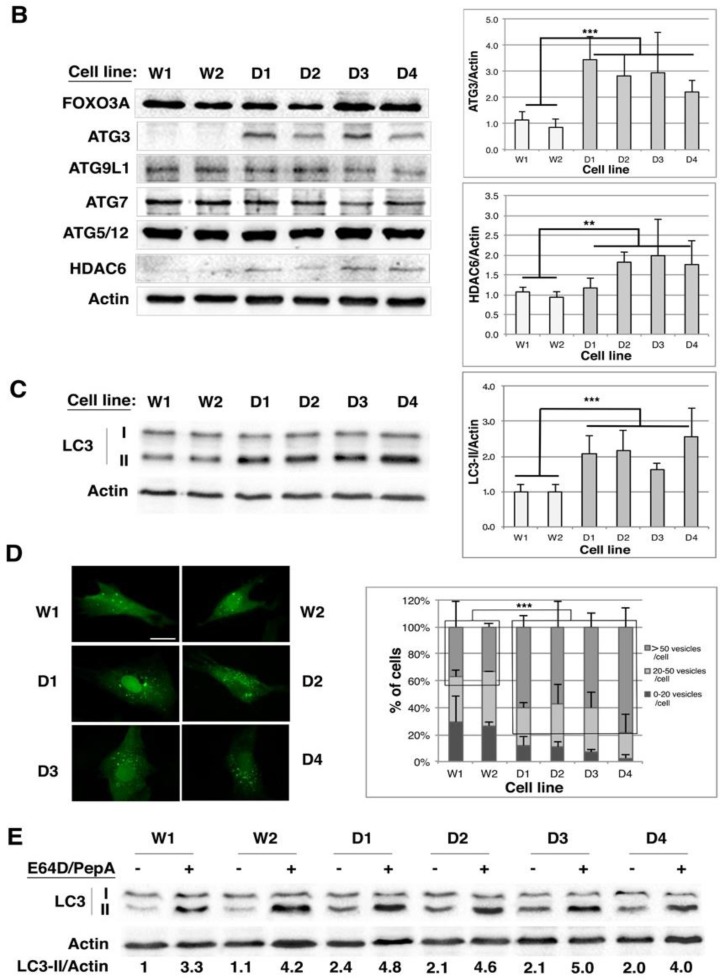
The autophagic flux is increased in DMD myoblast cell lines. (**A**–**C**) 10 µg of total protein extracts from control (W1 & W2) and DMD (D1 to D4) cell lines were separated by SDS-PAGE and analyzed by immunoblots probed with antibodies directed against (**A**) proteins involved in the initiation phase of the autophagic process: PI3K class III, BECN1 and BCL2; (**B**) proteins involved in autophagosome nucleation/elongation or transport/fusion: FOXO3a, ATG3, ATG9L1, ATG7, ATG5/12, HDAC6; (**C**) LC3-I and its lipidated form LC3-II. Actin is used as a loading control. The histograms show means ± SD of the normalized PI3KIII/Actin, ATG3/Actin, HDAC6/Actin and LC3-II/Actin ratios as described in Figure 2 (*n* = 3); (**D**) Control and DMD cell lines were transiently transfected with pEGFP-LC3; 24 h after transfection cells were fixed and analyzed with a fluorescence microscope (scale bar: 50 µm). The histogram shows quantification of the number of autophagic vesicles and indicates a statistically significant increase of cells containing more than 50 vesicles (*n* = 3); (**E**) Wild type and DMD cell lines were treated (+) or not (−) with a cocktail of lysosomal protease inhibitors (E64D and Pepstatin A) during 19 h. 10 µg of total protein extracts of each cell lines were separated by SDS-PAGE and analyzed by immunoblot using a specific antibody against LC3. This Western bot is representative of three identical experiments; after quantification, the LC3-II/Actin ratios was set at 1.0 for non-treated conditions in W1 cell line. ** *p* < 0.01, *** *p* < 0.001, one way ANOVA parametric test.

To assess autophagic activity in DMD cell lines we first quantified the levels of various proteins involved in the autophagic nucleation phase. We detected a 1.5–2-fold increase of class III PI3K level in DMD cell lines compared to control cells, whereas beclin1 and bcl2 levels remained unchanged (Figure 6A and Figure S5). As for proteins involved in the elongation phase (FOXO3a, ATG3, ATG9L1, ATG5-12 and ATG7), the closure of autophagosomes and their fusion to lysosomes (HDAC6), we observed a significant increase of ATG3 (2.2- (D4) to 3.4-fold (D1)) and of HDAC6 levels (between 1.8- and 2-fold in DMD2, 3 and 4 cell lines), which would be in favor of an enhancement of autophagosome formation (Figure 6B). We thus quantified LC3 levels and observed a 1.4–2.4 increase of the lipidated form of LC3 (LC3-II) in DMD cell lines compared to controls (Figure 6C). These results were confirmed with immunofluorescence analysis of control and DMD cell lines transiently expressing EGFP-LC3 (Figure 6D). Indeed, whereas 35% of control cell lines contained more than 50 autophagosomes per cell, this percentage raised up to 57% (D2) or 78% (D4) in DMD cell lines, indicating an increased number of autophagosomes in DMD cell lines. This increase could be the consequence of either increased autophagic flux or inhibition of the maturation/degradation step of autophagy. We thus performed LC3-II quantification in each cell line, in the presence or absence of E64D and pepstatin A, two inhibitors of lysosomal cathepsins that are known to block autophagosome maturation into autolysosomes (Figure 6E). Addition of these inhibitors further increased the already elevated level of LC3-II in DMD cell lines, indicating that the autophagic flux is not blocked in DMD cell lines compared to controls. However, LC3-II accumulation in these conditions is less intense in DMD myoblasts (2.0–2.4-fold) than in wild types (3.0–3.8-fold), which suggest that the fusion step may be slightly altered.

Consequently, our results describe an increase of BAG3/HSPB8 complexes associated with a stimulation of the autophagic flux in DMD myoblasts, but with a slight decrease of the maturation step efficiency.

### 2.6. NFκB Activity Is Stimulated in DMD Myoblasts

**Figure 7:**
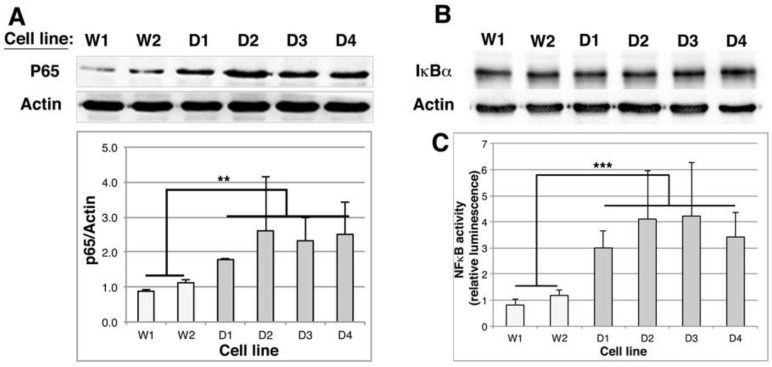
NFκB activity is stimulated in DMD myoblasts. (**A**,**B**) 10 μg of total protein extracts of Wild Type (W1 & W2) and DMD (D1, D2, D3 & D4) cell lines were separated by SDS-PAGE and submitted to an immunoblot hybridized with p65/RelA antibody (**A**) or IκBα antibody (**B**, *n* = 2). Actin is revealed as a loading control. In (**A**), the histogram shows the means ± SD of normalized p65/Actin ratios as described in Figure 2 (*n* = 3); (**C**) Cells were transiently transfected with pNFκBluc vector. The day after, the luciferase activity was measured. Results were normalized with control cells (ratio of luciferase activity in each cell line versus mean luciferase activity of control cells (*n* = 4). ** *p* < 0.01, *** *p* < 0.001, one way ANOVA parametric test.

Various stress conditions leading to proteasome inhibition, to increased BAG3/HSPB8 complexes and increased autophagic activity have been described to be associated with enhanced NFκB activity [17,41,42]. We thus checked whether this was the case in DMD cell lines. By Western blot, we first quantified RelA/p65 level, which is the transcriptional subunit of NFκB (Figure 7A) and observed a 1.8 (D1) to 2.6 (D3) increase of p65 subunit in DMD cell lines compared to control cells. In contrast, the level of IκBα, the inhibitory subunit of NFκB remained unchanged in control and DMD cell lines (Figure 7B). Control and DMD cell lines were thus transiently transfected with pNFκBluc reporter vector to quantify NFκB activity (Figure 7C). The luminescence produced was up-regulated by 3 (D1) to 4.2 (D3) fold in DMD cell lines in comparison to control cells, indicating that NFκB basal activity is increased in DMD myoblasts.

Taken together, our results demonstrate that DMD myoblasts are under protein aggregation stress as evidenced by the accumulation of protein aggregates, the modulation of expression of HSPB chaperones, the stimulation of NFκB activity, the altered proteasome activity associated with a BAG1 to BAG3 expression switch. This ensures up-regulation of BAG3/HSPB8 complexes and stimulation of an autophagic process albeit partially defective in its maturation step.

## 3. Discussion

In muscle cells, protein quality control (PQC) must be carefully regulated, in order to ensure optimal muscle efficiency, as evidenced by various studies highlighting the importance of PQC mediators in different types of muscular disorders. For instance, increases of molecular chaperone levels were detected in some muscles of *mdx* mice [28] or in oculopharyngeal muscular dystrophies [45]. As for the proteasome, an increase of its expression, of ubiquitin conjugation to muscle proteins, of transcripts encoding ubiquitin, of Ub-conjugating enzymes (E2) and of Ub-ligases (MURF1 and MAFBx/Atrogin-1) were reported to be associated with muscular dystrophies or atrophy [46,47], while deletion of the proteasome component Rpt3 was described to contribute to myofiber degeneration [48]. Autophagy defects have been described in DMD, Ullrich congenital muscular dystrophies (UCMD) and Emery–Dreifuss muscular dystrophies (EDMD) [20,30], while autophagy hyperactivation was observed in merosin-deficient congenital muscular dystrophy [49]. It is thus clear that PQC efficiency is modified in muscular disorders including DMD. However, it is still unknown whether and how PQC contributes to dystrophin-dependent muscle degeneration. Although numerous animal models for DMD have been developed (*C. elegans*, drosophila, zebrafish, mouse, rats, cats, dogs and pigs) [50,51,52] to investigate the physiopathology of DMD, all the studies have been carried out on mature, degenerating muscles; therefore, whether alterations, in particular of PQC mechanisms, could pre-exist at myoblast stage and may affect the regenerative capacities of the precursors, are still unknown. We thus decided to perform an exhaustive study of PQC actors in a unique DMD model consisting of transformed human DMD myoblast cell lines.

We first took advantage of transformed myoblasts to address the controverted question of dystrophin expression in non-differentiated muscles, which was either reported to be positive [53] or negative [54]. Western blot analysis of dystrophin with mandra1 antibody recognizing the C-terminal part of the protein but did not detect any signal in DMD myoblasts, which is in agreement with the dystrophin gene mutations present in DMD cell lines. Indeed, D1–D3 cell lines contain a premature stop codon in exon 41, which leads to premature protein translation termination. The D4 cell line contains a large out-of-frame deletion of the entire exon 44 leading to inclusion of aberrant amino acids, which generally leads to premature truncation of translation. The resulting dystrophins are thus non-functional and generally degraded [55]. However, we were able to detect dystrophin expression in undifferentiated control myoblasts. This expression is lower than in differentiated myotubes, but clearly confirms that dystrophin is expressed at the myoblast stage, which validates immortalized DMD myoblasts as powerful tools to study the involvement of PQC in the physiopathology of DMD.

In this study, we describe for the first time the existence of a protein aggregation stress in dystrophin-deficient myoblasts. Indeed, a protein aggregation of specific mutated dystrophin with missense mutations in actin binding domain 1 was reported [56], however, a global increase of protein aggregation level was, to our knowledge, never reported. Our results thus suggest that a PQC deficiency could be responsible for this accumulation of protein aggregates. HSPs are the first line actors of PQC, since they can refold misfolded/aggregated proteins. In contrast with the observation of increased levels of HspA and HspC proteins in muscle biopsies from DMD patients [57,58] or *mdx* hind limb muscle [29], we could not detect any modulation of these HSPs in human transformed DMD myoblasts; however, in these previous studies, HSPA and HSPC increases were mostly detected in regenerating muscle fibers [57], which is a different stage than undifferentiated muscle cells. The HSPB family are of special interest in muscles since they play a protective role in the maintenance of the cytoskeletal network and contractile elements [38,39,59,60]. In addition, abnormalities of some HSPB members are involved in muscle disorders: HSPB5 mutations are associated with myofibrillar myopathies [61] and HSPB1 or HSPB8 to distal myopathy and Charcot–Mary–Tooth disease type 2 [62,63]. In this study, we observed a drastic increase of HspB5 in DMD cell lines, which is consistent with the observation of increased levels of HspB5 in soleus and interosseous muscles of *mdx* mice [28]. In addition, we detected an accumulation of HspB5-containg aggregates in DMD myoblasts. Since we did not detect any disturbance of actin, tubulin or vimentin networks in DMD myoblasts, our results suggest that HspB5 chaperone activity might nevertheless be important for stabilizing the cytoskeletal network in a weakened dystrophic muscle and/or for holding misfolded muscle proteins in a folding-prone state and prevent their probable aggregation. Of interest, we also report for the first time a diminution of HSPB8 levels in DMD myoblasts that is, however, associated with an increase of HSPB8-containing aggregates suggesting that HSPB8 is specifically and efficiently redirected to protein aggregates, like HspB5. This increased recruitment of HSPB5 and HSPB8 to the aggregates associated with an accumulation of protein aggregates in DMD myoblastic cell lines suggest that the refolding capacities of the chaperone network might be overwhelmed and that protein degradation mechanisms could, thus, also be modulated.

The ubiquitin proteasome system (UPS) has been shown to play an important role in muscle protein catabolism by participating in disassembly or degradation of myofibrillar proteins or regulation of myogenesis [64]. In this study, we observed a 50% decrease of the chymotrypsin-like activity of the 26S proteasome in DMD myoblasts compared to control cells. Interestingly, site-directed mutagenesis in yeast allowed to identify the β5 (chymotrypsin-like) sites of the proteasome to be the most important sites for protein breakdown [65]. Our results suggest that down-regulation of the chymotrypsin-like activity could lead to decreased degradation rates of proteasome substrates in DMD myoblasts. This is quite surprising since increased levels of proteasome were detected in skeletal necrotic fibers of DMD patients [47]. Moreover, the use of proteasome inhibitors in *mdx* mice was found to improve the histo-pathological signs of the disease even if this treatment did not rescue every explant of DMD muscle biopsies from patients [20,66,67]. In addition, increased proteasome levels [47] and increased proteasome activity [66] were observed in muscles biopsies from DMD patients. This discrepancy could be linked to the differentiation state of the samples analyzed, myoblasts vs degenerating myotubes, or to the type of proteasome and activities measured; indeed, immunoproteasome content was described to be increased in dystrophic muscles of *mdx* mice whereas the total content of proteasome was unchanged [68]. The absence of modulation of the MuRF1 and MAFbx/atrogin-1 levels, two major muscle E3 UB ligases observed in DMD myoblasts was in accordance with quantifications performed in muscles biopsies of DMD patients [66]. Finally, we observed a decrease of BAG1-M level in DMD myoblasts in comparison to controls. BAG1 exists as multiple isoforms, BAG1-L, -M, BAG1 and BAG1-S, generated by alternative translation initiation [69]. Interestingly, BAG1-M isoform was reported to bind to HSPA1/A8, to the proteasome and to the CHIP Ubiquitin ligase [70,71], which suggest that a down-regulation of its level could decrease the efficiency of multi-ubiquitinated misfolded protein addressing to the proteasome.

Another member of the BAG family, BAG3, is highly expressed in skeletal muscle cells; its mutation being associated with severe myofibrillar myopathy [72]. Interestingly, BAG1 and BAG3 were found to be reciprocally regulated in aggregation-prone environments such as cellular aging or proteasome inhibition, inducing a switch from high BAG1/BAG3 ratio associated with BAG1-mediated proteasomal degradation to low BAG1/BAG3 ratio, associated with BAG3-mediated autophagy [40,41]. This selective autophagy also involves HSPB8, which complexed to BAG3, was described to clear aggregated proteins such as Htt43Q, SOD1G85A or filamin C in human skeletal muscles submitted to resistance exercise [17,43,44]. Our observation of increased BAG3 levels in DMD myoblasts associated with a detection of more numerous BAG3/HSPB8 complexes in DMD cells fit with these observations and allow us to report for the first time the existence of a BAG1 to BAG3 switch between control and DMD myoblastic cell lines, which suggest a shift to autophagy as a preferential protein degradation pathway. This was confirmed by our observations of increased levels of nucleation (class III PI3K), elongation (ATG3, LC3-II) or closure and transport (HDAC6) actors of autophagy/aggrephagy associated with an increase of autophagosomes in DMD myoblasts. Addition of lysosomal protease inhibitors allowed us to determine that the autophagic flux is not blocked in DMD myoblasts. However, the less intense accumulation of LC3-II in DMD cell lines compared to controls could be an indicator of a less efficient maturation step. We thus describe here, for the first time, a BAG1 to BAG3 switch associated with up-regulation of autophagic actors, increased numbers of autophagosomes but partial deficiency of the autophagosomes maturation and impaired degradation by the proteasome in DMD myoblasts. Such a mechanism was also recently described in a knock-in mouse model of spinal and bulbar muscular atrophy (SBMA), where changes in the expression ratios of BAG1 to BAG3 were associated with a shift from proteasome to autophagy degradation pathways [73]. Inhibition of autophagy flux was reported in *mdx* mice, with persistent activation of the AKT, mTOR axis, increased of p62 levels and decreased LC3 conjugation to phosphatidylethanolamine [30,74]. These results were nevertheless discordant with another study in the same mouse model reporting no differences in phosphorylated AKT and mTOR or LC3 levels between wild type and *mdx* mice, but an impairment of autophagy induction by starvation [75]. As for DMD patients, increased levels of phospho-AKT and p62 were observed in muscle biopsies of five patients [30]. Of interest, a recent study described increased autophagic actors (beclin1, ATG5-ATG12, class III PI3K) in dystrophic muscles of *mdx* mice, but decreased lysosomal content during disease progression, which seems consistent with our observations [76].

Since various stress conditions leading to proteasome inhibition or overload are described to be associated with enhanced NFκB transcription factor activity, and since NFκB can stimulate HSPB8 and BAG3 expression [42,77], we checked NFκB activity in control and DMD myoblasts and observed an increased level of the RelA/p65 subunit of NFκB, whereas the level of IκBα, the inhibitory subunit of the transcription factor was not modified. As a consequence, we observed increased NFκB basal activity in DMD myoblasts. These results are in accordance with studies reporting increased levels of p65 and NFκB activity, associated with stimulation of inflammatory pathways, in the muscles of infants soon after birth, prior to the onset of clinical manifestations [78,79]; interestingly, NFκB was also reported to be activated in dystrophic muscles of *mdx* mice. However, its stimulation was not associated with any phosphorylation or degradation of its inhibitory subunit IκBα, suggesting that the classical signaling pathway triggered by inflammatory cytokines is not responsible for this activation [80], which is consistent with our observations and the mechanisms of NFκB activation upon aggregation stresses [42,77,81]. Whether NFκB activation associated with protein aggregation stress is responsible for induction of inflammatory cytokines in DMD myoblasts should be the subject of further investigations.

In conclusion, in this study we report for the first time that the absence of dystrophin in transformed DMD myoblasts is accompanied by increased levels of multi-ubiquitinated proteins and p62-containing aggregates. Furthermore, this protein aggregation stress is associated with increased basal activity of NFκB transcription factor, BAG1 to BAG3 switch and up-regulation of BAG3/HSPB8 complexes that ensure preferential routing of misfolded/aggregated proteins to stimulated autophagy rather than to 26S proteasome. Thus, our results highlight that PQC modulation already pre-exists in non-differentiated DMD muscle cells. Consequently, modulation/restoring of this altered PQC could improve myoblasts and, thus, myotube physiology and might represent a valuable strategy for DMD therapies.

## 4. Materials and Methods

### 4.1. Cell Culture and Cell Lines

The cell lines used were derived from satellite cells that were isolated from muscle biopsies of healthy donors or Duchenne Muscular Dystrophy patients according to institutionally approved protocol and parents or legal representatives gave their written informed consent (protocol registered at the Ministère de la Recherche and Cochin Hospital Cell Bank, Paris, agreement No. DC-2009-944) and differentiated into myoblasts. These myoblasts were transformed with viral transduction of CDK4 (cyclin-dependent kinase-4) and hTERT (human telomerase reverse transcriptase) that are required to overcome cellular senescence as previously described [32]. Various clones were isolated and amplified; the cell lines were cultured at 37 °C in a 5% CO_2_ atmosphere in Skeletal Muscle Cell Growth medium (PromoCell, Heidelberg, Germany), complemented with 20% FCS (Invitrogen, Carlsbad, CA, USA), 0.2% dexamethasone (D4902, Sigma, St. Louis, MO, USA) and 1 μg/mL puromycin (selection marker; p8833, Sigma). Six distinct cell lines have been used in this study: (W1) and (W2) that are two cell lines derived from biopsies of healthy donors of 95 months and 121 months, respectively; 4 cell lines derived from the biopsies of 147 (D1, D2 and D3) and 142 (D4) months old patients: D1, D2 and D3 in which the dystrophin gene is interrupted by a STOP codon in exon 41 (c5758 C > T); D4 cell line, in which the exon 44 of the dystrophin gene is deleted (c.44 del from intron 43 (position 27,742) to intron 44 (position 116,255)).

### 4.2. Reagents and Plasmids

E64D (#E3132), Triton-X100 and Hoechst 33258 were from Sigma. Pepstatin A was from Merck-Millipore (Burlington, MA, USA). Bovine Serum Albumin was from Euromedex. Rabbit polyclonal antibody against ATG9L1 was from Abgent (AP1814a) (San Diego, CA, USA). Rabbit polyclonal antibodies against Bag3 (#ABC277) and p65 (#06-418) and mouse monoclonal antibody against actin (#MAB1501) were from Millipore. Mouse monoclonal anti-HSPB8 (#H00026353) was from Abnova (Taoyuan City, Taiwan). Mouse monoclonal antibodies against DNAJB1 (#SPA-450), HSPA1/A8 (#ADI-SPA-822F) and HspB5/alphaB-crystallin (#ADI-SPA-222F), rabbit polyclonal antibody against HspB5/alphaB-crystallin (#SPA-223) and rat monoclonal antibody against HSPC2/C3 (#ADI-SPA-835F) were from Enzo Life Sciences (Lausen, Switzerland). Rabbit polyclonal anti-LC3B (#L7543) and mouse monoclonal anti-acetylated α-tubulin (#MABT868) were from Sigma. Rabbit polyclonal antibodies against Beclin1 (#ab51031), HDAC6 (#ab133493) and Pericentrin (#ab4448) and mouse monoclonal antibody against Atg3 (#ab56409) and VCP (#ab11433) were from Abcam (Cambridge, UK). Mouse monoclonal anti-multi-ubiquitin (clone FK1; #D071-3) was from MBL. Goat polyclonal antibody against HspB1/Hsp27 (#sc-1190) and mouse monoclonal anti-dystrophin (MANDRA1; #SC-73592) were from Santa Cruz Biotechnology, Inc. (Dallas, TX, USA). Anti-NBR1 is a rabbit polyclonal antibody from PTGLab (#16004-1-AP). Anti-Atg5 (#2630), anti-PI3K III (#3811) and anti-αtubulin (#2144) rabbit polyclonal antibodies were from Cell Signaling (Danvers, MA, USA). Mouse monoclonal antibody against vimentin was from DAKO (#M0725). Mouse monoclonal antibody against p62 was from BD Sciences (#610832). Anti-MHC monoclonal antibody (#MAB4470) was from R&D Systems. Goat anti-mouse (#170-6516) and goat anti-rabbit (#170-6515) secondary antibodies were from Bio-Rad (Hercules, CA, USA). Donkey anti-goat (#sc-2020) secondary antibody was from Santacruz. Rabbit anti-rat secondary antibody (#A5795) was from Sigma. Goat anti-mouse Alexa Fluor 488 (#A11001) or 568 (#A11031), goat anti-rabbit Alexa Fluor 488 (#A11034) or 568 (#A11011) secondary antibodies were from Thermofischer (Waltham, MA, USA). pNFκB-LUC was from Clontech (Mountain View, CA, USA). pGL3 promotor vector was from Promega (Madison, WI, USA). pEGFP-LC3 was a kind gift from T. Yoshimori (Research Institute for Microbial Diseases, Tokyo, Japan). 

### 4.3. Transfection

Twenty four hours before transfection, myoblasts were seeded at a density of 10^6^ cells/100 mm dishes or 5.8 × 10^5^ cells/60 mm dishes. Cells were then transiently transfected with the desired plasmid (pNFκBluc vector or pGL3-promotor vector) by using the Jetprime^®^ transfection reagent (Polyplus, Illkirch-Graffenstaden, France), according to the supplier’s protocol.

### 4.4. Gel Electrophoresis and Western-Blot

Briefly, 10 µg of total protein extracts were separated by SDS-PAGE on acrylamide/bisacrylamide (Euromedex) or 3–15% gradient Tris-Acetate gels, in Tris-Glycin 1× 0.1% SDS buffer or in Tris-Acetate SDS Running Buffer [82]. After electrophoresis, proteins were transferred onto Protran BA85 nitrocellulose (Perkin Elmer, Waltham, MA, USA) or immobilon-P (Millipore) membranes and blots were incubated with primary antibodies and horseradish peroxidase-conjugated secondary antibodies. Revelation was performed with ECL detection reagents (Clarity Western ECL substrate from Bio-Rad or ECL blotting detection reagent from Amersham). Western blot imaging was performed with Chemidoc MP (Bio-Rad) based on charge-coupled device detection technology. Image capture and analysis of Western-blot were processed by ImageLab 4.0 software and quantification by Image J software (NIH). 

### 4.5. Proteasome Assay

The control and DMD myoblasts cell lines were plated in 96-well culture plates at a density of 10^4^ cells/well. The day after transfection, proteasome activities (caspase-, trypsin- and chymotrypsin-like activities) were measured using cell-based Proteasome-Glo™ assay (Promega) as previously described [42]. The proteasome inhibitors MG132 and lactacystin (10 µM, 2 h) were used as negative controls. Luminescence was measured using a Victor3 Luminometer (Perkin Elmer). The relative light units produced were reported to 50 µg of total proteins.

### 4.6. Luciferase Assay

Myoblasts, transiently transfected with pNFκBluc vector the day before, were either untreated or submitted to a 90-min heat shock at 43 °C. 24 h after the transfection, the cells were resuspended into PBS and luciferase activity was quantified using the steady-glo^®^ luciferase assay (Promega) as previously described [17]. Luminescence was measured as described above and related light units produced were reported to 1 µg of total cellular proteins.

### 4.7. Luciferase Refolding Assay

This technic was previously described [83]. Briefly, myoblast cell lines transiently transfected with pGL3-promotor vector were submitted to a 30-min heat shock at 43 °C to inactivate luciferase. Then cells were incubated at 37 °C to allow luciferase refolding and cell samples were taken at various time points (0.5–4 h) for luciferase activity measurements. Luminescence was reported to 1 µg of total cellular proteins.

### 4.8. Filter Trap Assay

SDS-insoluble aggregates were analyzed by filter trap analysis as previously described [17]. Briefly, cells were scraped in 2% SDS-FTA buffer (FTA: 150 mM NaCl, 50 mM DTT, 10 mM Tris-HCl, pH 8). Next, samples were homogenized by passages through 25 G needle. 2.5 µg of protein extracts were diluted by a factor of 2–8 and applied into a slot blot apparatus onto a protran BA83 nitrocellulose membrane (Schleicher and Schuell, Dassel, Germany) pre-washed with 0.1% SDS-FTA buffer. Then the membrane was washed with 0.1%SDS-FTA buffer and 0.1%Tween-TBS buffer (TBS: 20 mM Tris-HCl, pH7.6, 137 mM NaCl) and processed for immunoblotting. Filter trap assay allows quantification of protein aggregates by looking for similar intensities of revealing between the various slots of diluted protein extracts of each cell line.

### 4.9. Fluorescence Microscopy Analysis

Cells were grown on glass coverslips or on polymer µ-slide IbiTreat (Ibidi). Thereafter, cells were fixed during 10 min with methanol, permeabilized and saturated with PBS-0.2%Triton-2% BSA and hybridized with various primary antibodies (p62, vimentin, desmin, actin, HspB5, MyHC) and Alexa Fluor secondary antibodies. Hoechst 33258 reagent was used to stain nuclei (5 min, 1 ng/mL). Observations were performed on Zeiss Axio Imager Z1 photomicroscope (Zeiss Inc., Oberkochen, Germany). Images were digitized with a camera (Coolsnap HQ2; Roper scientific) and acquired with Metavue Imaging system. Digitalization was done with Metavue software; images adjustments were performed on ImageJ. 

### 4.10. Proximity Ligation Assay (PLA)

PLA is an antibody-based method in which two proteins are immunolabeled first with two primary antibodies and then with different species-specific secondary antibodies conjugated to complementary oligonucleotides. When two antibody molecules are in close proximity, the complementary DNA strands can be ligated, amplified and visualized with a fluorescent probe as distinct puncta. Each spot may represent a single complex containing each of two interacting proteins. 105 cells were seeded on glass coverslips in 35 mm cell culture dishes and were fixed and permeabilized as described above. Mouse monoclonal anti-HSPB8 and rabbit polyclonal anti-BAG3 were used for detecting BAG/HSPB8 complexes with Duolink^®^ In Situ Orange kit mouse/rabbit (Sigma-Aldrich) according to the manufacturer instructions. Images of immunostaining were captured on a Zeiss confocal laser-scanning microscope LSM800 (63× objective). Digitalization was performed with Zen software. Automated counting of dots in 50 cells of each cell line was performed with Fiji’s particle analysis after running through the watershed program.

### 4.11. Statistics

One-way ANOVA parametric tests were performed to compare mean values inside the wild type group or DMD group after verifying homoscedasticity by F tests and showed no statistical differences inside each group. One-way ANOVA parametric test was also applied to compare the mean values of all Wild Type cell lines to the mean of all DMD cell lines results (* *p <* 0.05; ** *p <* 0.01; *** *p <* 0.001).

## Supplementary Materials

Supplementary materials can be found at www.mdpi.com/1422-0067/19/1/178/s1.

## Abbreviations

DMD	Duchenne Muscular Dystrophy
HSP	Het Shock Protein
MyHC	Myosin Heavy Chains
PQC	Protein Quality Control
Ub	Ubiquitin
UPS	Ubiquitin Proteasome System
