# Supplementary material for: Modulation of Protein Quality Control and Proteasome to Autophagy Switch in Immortalized Myoblasts from Duchenne Muscular Dystrophy Patients
Source: Int J Mol Sci. 2018 Jan 7;19(1):178. doi: 10.3390/ijms19010178 (PMC5796127; doi:10.3390/ijms19010178)
Supplement: Supplementary file 1 [file ijms-19-00178-s001.pdf]

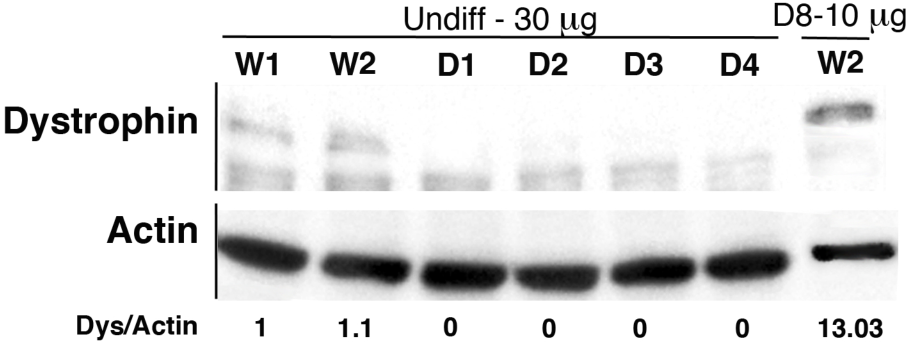

Figure S1: Characterization of dystrophin expression in human immortalized myoblasts. 30 µg of total protein extracts from undifferentiated Wild Type (W1 & W2) and DMD (D1 to D4) cell line were separated by SDS-PAGE. Analysis of Dystrophin expression was performed by immunoblotting thanks to the use of Mandra1 antibody. Actin is used as a loading control. As a positive control, 10 µg of total protein extracts of 8 days differentiated W2 cell line (D8, W2) were submitted to the same protocol (n=2).

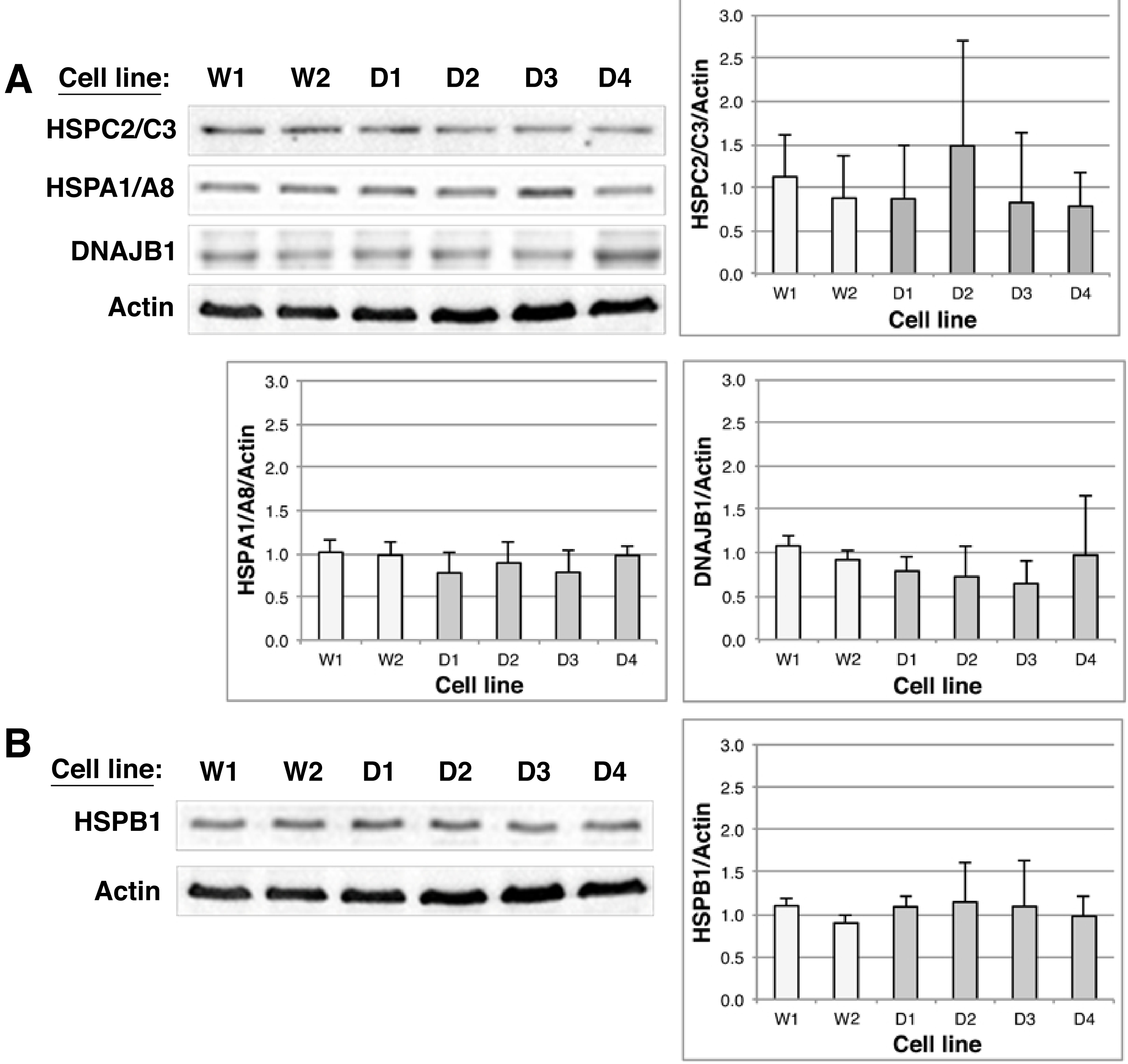

Figure S2: Foldases and HSPB1 holdase expression are not modulated in human immortalized DMD myoblasts. (A and B) 10 µg of total protein extracts from Wild Type (W1 & W2) and DMD (D1 to D4) cell lines were separated by SDS-PAGE. Heat Shock proteins expression was analyzed by immunoblotting using (A) anti-HSPC2/C3, -HSPA1/A8, -DNAJB1 and -Actin antibodies; (B) anti-HspB1 and -Actin. Histograms indicate HSPC2/C3/Actin, HSPA1/A8/Actin, DNAJB1/Actin and HSPB1/Actin ratios. No statistically significant differences were observed between control and DMD cell lines (ANOVA, n=3)

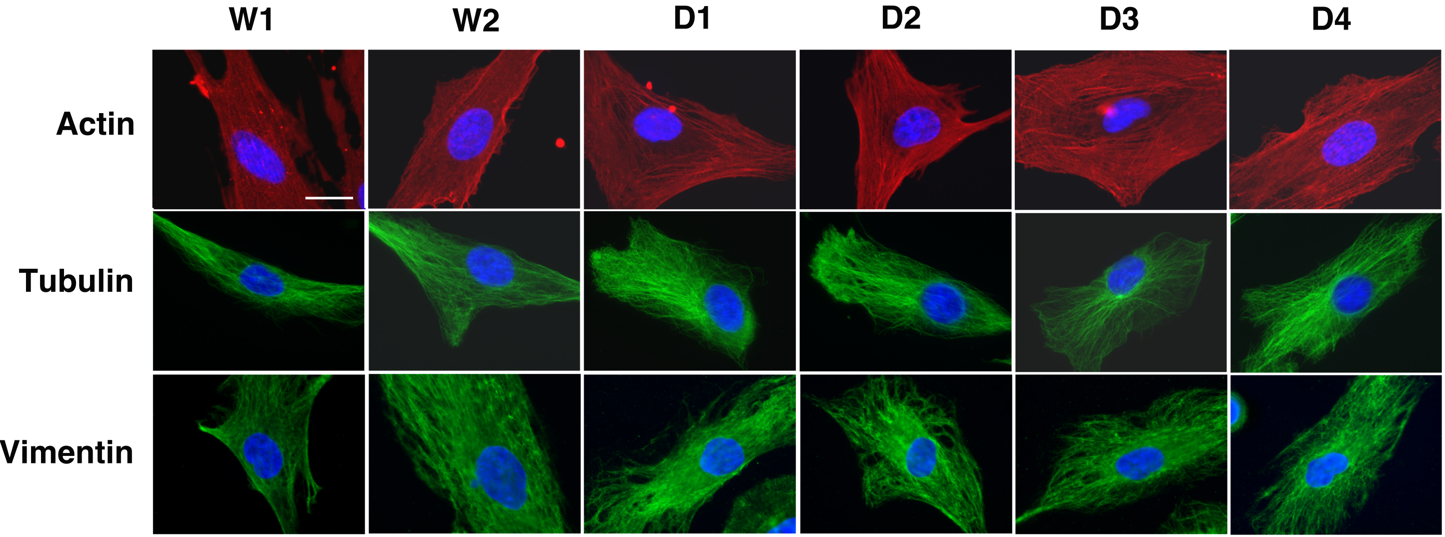

Figure S3: Cytoskeleton filaments are not altered in DMD myoblasts. Wild Type (W1 & W2) and DMD (D1 to D4) cell lines were fixed, permeabilized and stained with antibodies against, actin (red), α-tubulin (green) or vimentin (green). Nuclei were stained with Hoechst (blue). Cells were analyzed with a fluorescence microscope (scale bar: 50 μm) (n=3).

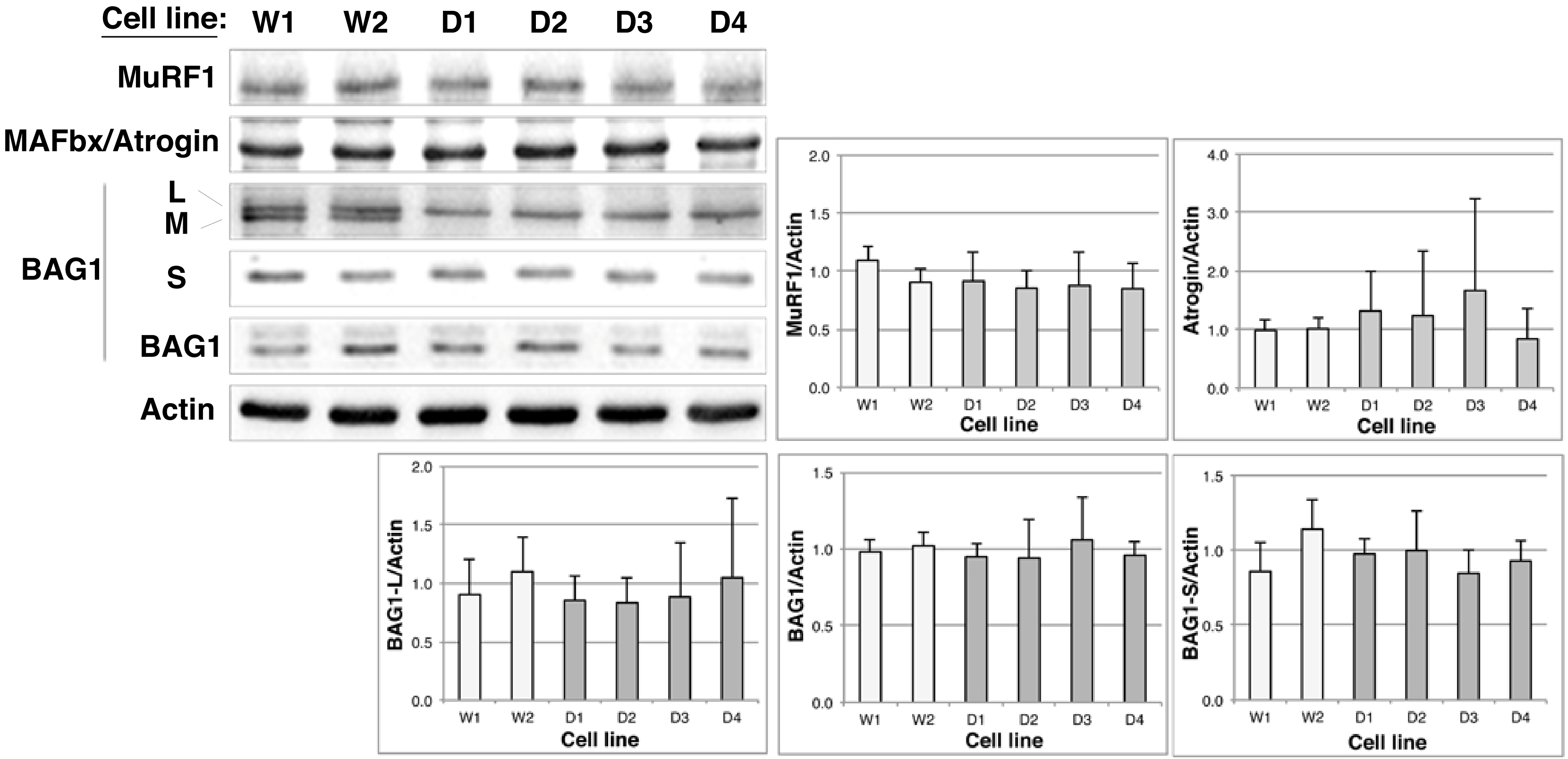

Figure S4: E3 Ub ligases, BAG1, BAG1-L and BAG1 expression is not modulated in DMD myoblasts. 10 µg of total protein extracts of Wild Type and DMD cell lines were separated by SDS-PAGE. Analysis of the expression of the E3 Ub ligases MuRF1 and MAFbx/Atrogin and of BAG1 isoforms expression was performed using specific antibodies. Actin is revealed as a loading control. Histograms show the ratios of the various proteins *vs* Actin. No statistically significant differences could be observed (ANOVA test, n=3).

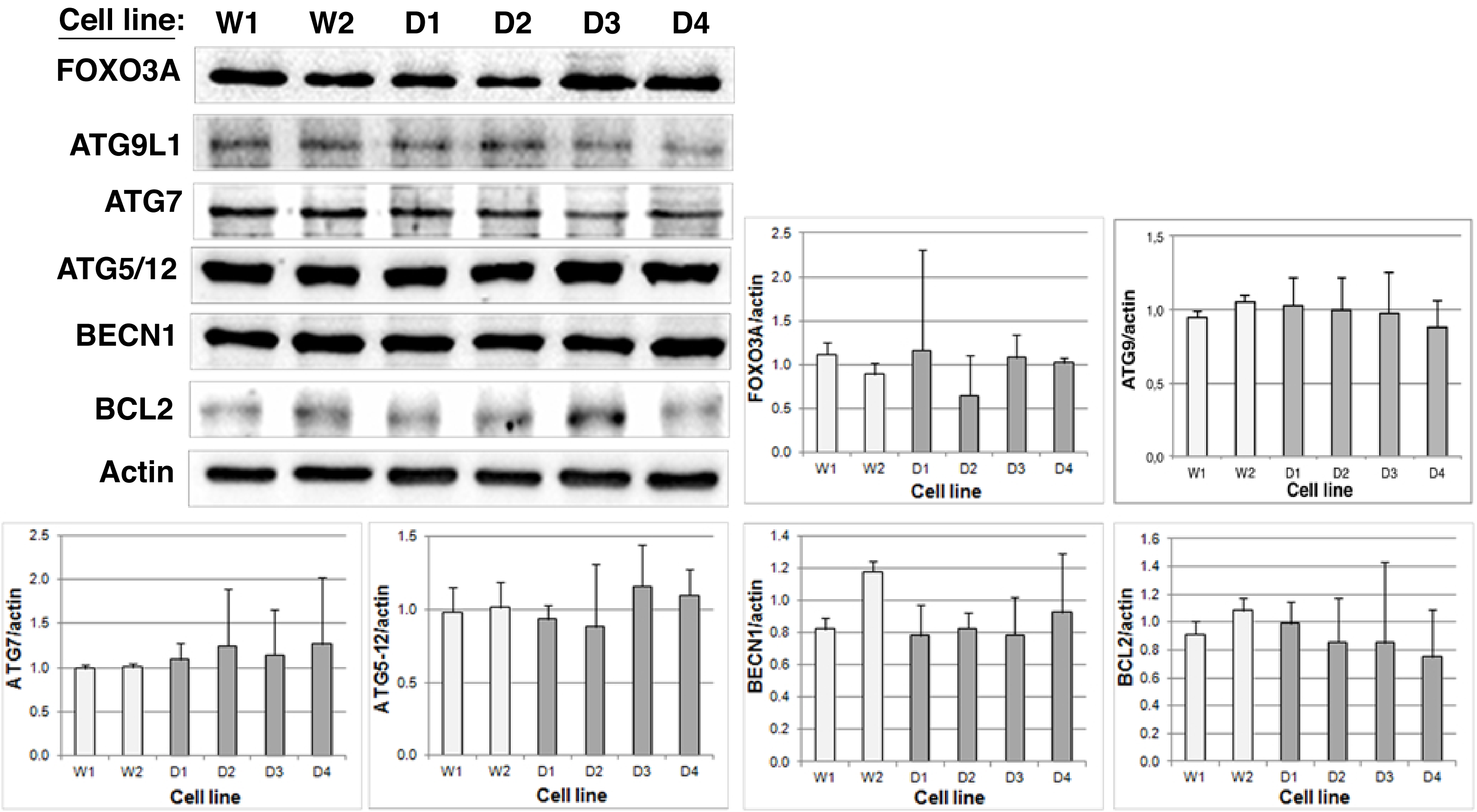

Figure S5: FOXO3A, ATG9L, ATG7, ATG5/12, BECN1 and BCL2 levels are not modulated in DMD myoblasts. 10 µg of total protein extracts of Wild Type and DMD cell lines were separated by SDS-PAGE. Analysis of the expression of FOXO3a, ATG9L1, ATG7, ATG5/12 BECN1, BCL2 and Actin was performed using specific antibodies. Actin is revealed as a loading control. Histograms show the ratios of the various proteins *vs* Actin. No statistically significant differences could be observed (ANOVA test, n=3).

Densitometry FTA DMD / Densitometry FTA WT		Cell line			
		D1	D2	D3	D4
MultiUb	W1	2.0	2.4	2.9	3.3
	W2	2.3	2.0	2.4	2.9
p62	W1	1.9	1.6	2.0	1.6
	W2	1.8	1.8	1.9	1.7
HSPB5	W1	3.8	3.6	3.6	5.2
	W2	3.9	3.9	3.8	3.8
HSPB8	W1	3.4	3.8	2.7	3.0
	W2	3.1	3.2	2.9	3.7
BAG3	W1	1.9	1.6	1.9	2.2
	W2	1.7	1.6	1.8	1.9

Table S1: Densitometric quantification of filter trap experiments.
2.5 µg of total protein extracts from WT and DMD cell lines were slot-blotted at four different dilutions (1, 1/2, 1/4 and 1/8) on a cellulose acetate membrane and probed with multi-ubiquitin, p62, HSPB5, HSPB8 or BAG3 antibodies. Densitometric analysis of the blots were performed and ratios between densitometric quantification of the 1/8 dilutions of DMD cell extracts and densitometric quantifications of the 1/8 dilutions of WT cell extracts were calculated and presented in the table. Standard deviation are less than 15%.
